# Update on the Management of Brain Metastasis

**DOI:** 10.1007/s13311-022-01312-w

**Published:** 2022-11-23

**Authors:** Karanvir Singh, Shreya Saxena, Atulya A. Khosla, Michael W. McDermott, Rupesh R. Kotecha, Manmeet S. Ahluwalia

**Affiliations:** 1 Division of Medical Oncology, Miami Cancer Institute, Baptist Health South Florida, Miami, FL 33176 USA; 2 Division of Neurosurgery, Miami Neuroscience Institute, Baptist Health South Florida, Miami, FL 33176 USA; 3 Division of Radiation Oncology, Miami Cancer Institute, Baptist Health South Florida, Miami, FL 33176 USA; 4 Herbert Wertheim College of Medicine, Florida International University, Miami, FL 33199 USA

**Keywords:** Brain metastases, Systematic review, Systemic therapy, Immunotherapy, Targeted therapy, Actionable mutations

## Abstract

Brain metastases occur in almost one-third of adult patients with solid tumor malignancies and lead to considerable patient morbidity and mortality. The rising incidence of brain metastases has been ascribed to the development of better imaging and screening techniques and the formulation of better systemic therapies. Until recently, the multimodal management of brain metastases focused primarily on the utilization of neurosurgical techniques, with varying combinations of whole-brain radiation therapy and stereotactic radio-surgical procedures. Over the past 2 decades, in particular, the increment in knowledge pertaining to molecular genetics and the pathogenesis of brain metastases has led to significant developments in targeted therapies and immunotherapies. This review article highlights the recent updates in the management of brain metastases with an emphasis on novel systemic therapies.

**Supplementary Information:**

The online version contains supplementary material available at 10.1007/s13311-022-01312-w.

## Introduction

Brain metastases (BM) affect up to one-third of adults with solid tumor malignancies and are associated with significant cancer patient morbidity, anxiety, and mortality. Approximately 70,000–400,000 patients will develop BM each year in the USA [1–3]. Consequentially, BM represent an important public health care burden that is also ten times more common than primary malignant brain tumors. The rising incidence of BM has partly been attributed to the availability of better imaging modalities (MRI), increased systematic screening for at-risk patients, and improved systemic therapies with extra-cranial control but limited intracranial protection as the central nervous system (CNS) is a sanctuary site [4, 5]. Despite the staggering incidence of brain metastases, cancer-specific incidence ratios are not well described in the literature.

As most systemic therapies had limited blood barrier penetration, the traditional practice was comprised of regional, brain-directed therapies including radiation therapy and surgical resection [6, 7]. However, recently, the paradigm has shifted to immunotherapy as a first-line choice for well-selected, asymptomatic patients with specific histologies and the use of targeted agents in oncogenic-driven tumors with actionable mutations [8–10]. Additionally, these novel therapies with CNS activity have also significantly contributed to the improved prognosis of BM patients [4, 11]. The purpose of this article is to highlight the treatment of BM with a specific focus on novel systemic therapy agents.

## Prognosis

Earlier studies estimating the prognosis of BM patients led to the development and application of various prognostic indices, such as the recursive partitioning analysis (RPA, a tiered prognostic system based on age, extra-cranial disease status, and the Karnofsky performance status) and the disease-specific graded prognostic assessment (DS-GPA) which is a more modern scoring system based on four objective risk factors [12–16]. The DS-GPA is based on accumulated data of brain metastases patients from several institutions and has recognized important prognostic factors within each major primary tumor site, including Karnofsky performance status (lung, melanoma, renal cell, breast, and gastrointestinal primaries), age (lung, breast), presence of extra-cranial metastases (lung), and the number of brain metastases (lung, melanoma, and renal cell) [12, 17–20]. More recent studies demonstrated differences in outcomes based on breast cancer, melanoma [20], and NSCLC subsets [21–23]. The most recent versions of the prognostic scoring systems have also now integrated tumor biology and molecular profiles, such as EGFR and ALK alterations in NSCLC adenocarcinoma (Lung-molGPA) [21], estrogen/progesterone and HER2-receptor status for breast cancer (Breast-GPA) [19], and BRAF status in melanoma (Melanoma-molGPA) [20] to more accurately estimate a modern BM patient’s outcome. It is important to make an accurate prognostication, as this guides efficient treatment decision making and identifies the population requiring an aggressive brain-directed therapy, as opposed to a palliative approach.

## Treatment

### Neuro-Oncology

Brain metastasis patients face neurologic symptoms from both underlying intracranial disease and treatment-related sequelae [22–25] such as symptoms related to vasogenic edema, seizures, venous thromboembolism, radiation necrosis, and neurocognitive decline [26, 27]. Often these symptoms require medication management, including corticosteroids, antiepileptic drugs, analgesics, and other supportive medications [28–31]. Routine prophylactic use of antiepileptic drugs is, however, not recommended [32]. Dexamethasone is the main glucocorticoid employed to reduce perilesional edema and inflammation, in the setting of symptomatic brain metastases [33]. Finally, bevacizumab, a monoclonal antibody targeting vascular endothelial growth factor (VEGF) has been explored as a steroid-sparing agent. It has been demonstrated to reduce radiation necrosis-associated capillary leakage and brain edema [34].

### Neurosurgery

Neurosurgical intervention plays a significant role in the management of brain metastases patients [35]. The craniotomy is typically performed in (1) newly-diagnosed patients without a known underlying primary, (2) BM-associated symptoms resistant to corticosteroids, (3) large metastases, and (4) solitary BM (i.e., one BM without extra-cranial disease) [36, 37]. Multidisciplinary management is the best alternative to the management of finding any brain metastases and may require a carefully evaluated methodology [38, 39].

In the case of a single BM, surgical resection is preferred over WBRT alone [1, 2]. The efficacy of surgery as compared to WBRT for the management of solitary BMs has been supported by two randomized clinical trials. Patchell et al. compared resection combined with WBRT with WBRT alone. This showed an increased survival (40 weeks vs 15 weeks), fewer local recurrences (20% vs 52%), and a better quality of life in patients undergoing resection with WBRT [6]. Vecht et al. found a longer overall survival in patients with a single BM, undergoing resection and WBRT (10 months vs 6 months) in addition to a longer period of functional independence [40]. Muacevic et al. conducted a phase III trial comparing WBRT and microsurgery in combination with gamma knife surgery alone and noted similar survival and local tumor control rates but improved quality of life metrics after radiosurgery alone (*p* < 0.05) [41]. Moreover, for the treatment of solitary brain metastases, Roos et al. compared surgery and radiosurgery, both with adjuvant WBRT, and a reported better median OS with radiosurgery and WBRT (6.2 vs 2.8 months, *p* = 0.20) [42].

### Radiation Therapy

The most common form of local therapy used in patients with BM is radiation therapy. There are multiple different options, including whole-brain radiation therapy (WBRT) with or without hippocampal avoidance (HA-WBRT), stereotactic radiosurgery (SRS), and brachytherapy.

#### WBRT—Whole Brain Radiation Therapy

WBRT is currently used for patients with leptomeningeal disease or those with multiple brain metastases (> 4 lesions) [43]. Conventional WBRT is associated with several short- and long-term side effects, such as fatigue, anorexia, xerostomia, nausea, and alopecia, as well as cognitive dysfunction, balance problems, and hearing loss [44]. Pharmacologic strategies have been developed to help mitigate these side effects. RTOG 0614, a randomized trial compared memantine (used for 24 weeks including a 4 week up-titration period), an N-methyl-D-aspartate (NMDA) receptor antagonist, against placebo among patients receiving WBRT [45]. This trial showed preservation in the delayed recall at 24 weeks (primary endpoint) and a significantly longer time to cognitive decline noted among patients receiving memantine [45]. Hence, memantine is a valuable adjunctive therapy for patients receiving WBRT. Radiotherapeutic advances, such as the use of hippocampal avoidance (HA-WBRT), have also demonstrated reductions in neurocognitive dysfunction [46–48]. Most recently, NRG CC001 compared these two strategies and demonstrated lower rates of cognitive failure with HA-WBRT and memantine compared to conventional WBRT and memantine, proving HA-WBRT and memantine as the standard in most patients lacking brain metastases in or around 5 mm of the hippocampi or leptomeningeal disease [48, 49].

#### SRT—Stereotactic Radiosurgery

SRS can be delivered as either a single fraction of extremely conformal, high-dose treatment (generally 18–24 Gy) or as moderately-dosed fractions termed fractionated SRS (FSRS) ranging from 24 to 27 Gy in 3 fractions, or 30 Gy in 5 fractions [50]. This is commonly used for patients with intact brain metastases and is supported for those with limited intracranial disease (1–4 lesions) [51] as well as select patients with multiple lesions (4–15 BM) [52, 53]. In addition, there is increasing interest in combining SRS with select systemic therapy agents to improve response rates and duration of disease control [54–56]. Given the significant paradigm shift to focal therapy alone for intact lesions, SRS is also increasingly being utilized around operable brain metastases as well [57]. Although no high-quality prospective data exists to compare primary SRS to surgery, retrospective data demonstrate higher rates of tumor control with surgery and SRS compared to SRS alone for patients with large brain metastasis (≥ 4 cc or 2 cm in diameter) [58, 59]. A randomized trial of post-operative SRS vs WBRT was associated with similar survival but the preservation of neurocognitive decline in those treated with focal therapy, supporting its use in patients with limited intracranial disease [60]. Although focal therapy following surgery is seemingly attractive in comparison to WBRT, there are also significant drawbacks to this approach, such as difficulties in target volume delineation, high rates of local failure, and the risk of leptomeningeal spread. Two strategies are underway to further improve patient outcomes with focal therapy. First, to increase to the dose to the resection cavity and local tumor control rates, fractionated SRS is often utilized in clinical practice. Prospective randomized trials are in fact currently underwent comparing post-operative SRSto FSRS (NCT04114981). Second, one can perform SRS prior to surgery, which is associated with an improved ability to delineate the target as well as a smaller treatment volume (intact brain metastasis vs post-operative cavity) and has been associated with high rates of disease control even for very large brain metastases [61]. Retrospective comparisons between pre-operative SRS and post-operative SRS for operable brain metastases have demonstrated similar rates of local recurrence (*p* = 0.24) and overall survival (*p* = 0.1) and reduced rates of leptomeningeal disease failure (3.2 vs 16.6%, *p* = 0.10) and radiation necrosis (4.9% vs 16.4%) with pre-operative SRS supporting this approach [59]. Current and future trials (NCT03750227 and NCT03741673) will compare pre-operative SRS vs post-operative SRS to provide prospective randomized evidence to guide clinical practice.

#### Brachytherapy

Brachytherapy allows for the placement of radioactive isotopes intraoperatively within a resection cavity, so highly conformal high-dose radiation is delivered to the targeted area with a limited dose to the remainder of the brain parenchyma [62]. Newer brachytherapy carriers have allowed for more uniform dose distributions and protection against the brain parenchyma and have resulted in a resurgence of the utilization of this technique, and ongoing registries are prospectively collecting clinical outcomes (NCT04427384). The brachytherapy sources used in modern practice consist of Cesium-131 (C-131) seeds, and prospective phase 1/2 trials have demonstrated impressive local disease control rates in patients with large (> 2 cm) newly-diagnosed brain metastases [63]. Ongoing trials are also comparing post-operative brachytherapy to post-operative SRS/FSRS in patients with large operable brain metastases (NCT04365374). Brachytherapy can also be used as an efficient salvage therapy for patients with locally recurrent tumors after prior radiotherapy [64]. Therefore, this provides an additional valuable resource for managing intracranial disease in operable patients with resectable BM.

### Systemic Therapy

Traditionally, systemic therapy agents had limited efficacy in CNS metastasis, and therefore, local brain-directed therapy was the treatment of choice for almost all patients [65]. However, this has changed substantially in recent years due to the advent of targeted and immunotherapy as detailed in the next sections.

#### Non-Small Cell Lung Cancer (NSCLC)

Systemic therapy in patients with NSCLC with brain metastasis is dependent on the presence or absence of targetable mutations. In the USA, about 33–45% of lung adenocarcinomas harbor such genetic changes. The number is significantly greater in nonsmokers [9, 65, 66]. Anti-PD1 agents like pembrolizumab [67] and nivolumab especially in PD-L1 positive patients or who harbor other biomarkers for immunogenicity, or anti-folate chemotherapeutic agent, pemetrexed [68, 69] in patients with adenocarcinomas may aid in controlling intracranial disease in some patients, although responses can be limited.

In NSCLC, EFGR mutations are quite common and occur in 15–30% of cases. Ninety percent of patients with EGFR alterations in NSCLC harbor exon 19 deletions or exon 21 L858R substitutions and are sensitive to EGFR-targeting TKIs. 1st, 2nd, and 3rd generation EGFR TKIs may prove helpful in uncommon EGFR mutations barring exon 20 insertions [70, 71]. Prospective trials for erlotinib, gefitinib, and afatinib in patients with EGFR alterations and brain metastases have documented 70–88% intracranial response rates [72–74]. Osimertinib, a 3rd generation inhibitor, is the present choice of treatment in brain metastases patients with EGFR-mutant lung cancer [75]. Initially, osimertinib had shown great promise with high intracranial response in patients with extracranial T790 resistance mutation who have received prior EGFR-TKI therapy [76, 77]. More recently, osimertinib has become the drug of choice for newly diagnosed EGFR lung cancer patients. The FLAURA study compared osimertinib with TKIs (gefitinib and erlotinib) that demonstrated increased progression-free interval and overall survival and an improved intracranial response with osimertinib (91% vs 68%, respectively) [73–75, 78, 79]. A dual inhibitor of EGFR and HER2, Neratinib may be helpful in patients with select EGFR mutations [80]. Studies to analyze tumor DNA in CSF may help explore CNS progression after the previous intracranial response [81]. ALK targeting therapies such as alectinib, ceritinib, lorlatinib, and brigatinib have demonstrated high intracranial disease control rates [65, 82–91]. Lorlatinib has proved helpful after the progression of symptoms with other ALK targeting therapies [88], but the side effects such as speech changes, mood changes, weight gain, peripheral neuropathy, and gastrointestinal effects have made its use as a first-line treatment challenging in ALK-rearrangement patients [92–94]. The LIBRETTO-001 trial evaluated the efficacy of selpercatinib, a specific RET inhibitor for BMs in patients with RET fusion-positive NSCLC. The iORR reported was 82%, and the median intracranial PFS was determined to be 13.7 months at a follow-up duration of 11.0 months [95]. Adding radiation to standard TKI therapy for NSCLC patients may prove beneficial, and there are trials exploring the combination studies [94]. Further randomized studies are needed to evaluate the role of combined modalities and sequencing of these therapies.

There has been tremendous excitement about the use of immunotherapy in NSCLC and brain metastases. A phase II study out of Yale Cancer Center by Goldberg et al. showed that pembrolizumab is effective in treating NSCLC with untreated brain metastases in PD-L1 expression of at least 1%. A total of 29.7% of brain metastasis patients in this cohort responded to pembrolizumab, and the median follow-up was 8.3 months [67]. The Checkmate 227 trial showcased an increase in overall survival in the nivolumab and ipilimumab group as compared to chemotherapy in patients with PD-L1 expression of > = 1% or < 1%. The 4-year survival rate was 29% versus 18% in PD-L1 > = 1% and 24% versus 10% in PD-L1 < 1% for nivolumab and ipilimumab versus chemotherapy, respectively [96]. The group with brain metastases derived benefit as well as patients who did not have brain metastases (Table 1).

**Table 1:** Summary of different drugs for brain metastasis

**Study**	**Drug**	**Patient population**	**N**	**CR**	**PR**	**SD**	**PD**	**ORR**	**OS (months)**	**PFS (months)**
AURA3 [76]	Osimertinib	419	30	7%	63%	23%	3%	70%	N/A	11.7
FLAURA [124]	Osimertinib	200	22	23%	68%	5%	0%	80%	38.6	18.9
ASCEND 4 [82]	Ceritinib	376	35	11%	60%	17%	6%	73%	N/A	10.7
NCT01801111 [83]	Alectinib	138	35	20%	37%	29%	9%	57%	N/A	8.9
ALTA [87]	Brigatinib	275	44	5%	48%	32%	N/A	71%	N/A	N/A
NCT01970865 [88]	Lorlatinib	276	81	20%	43%	25%	9%	51%	N/A	7.3
LANDSCAPE [98]	Lapatinib	45	44	5%	52%	36%	7%	66%	17.0	N/A
HER2CLIMB [102]	Tucatinib	291	55	6%	42%	44%	4%	47%	18.1	9.9
Break MB [125]	Dabrafenib	325	74	0	7%	27%	40%	39%	33.1	16.1

#### Breast Cancer

Most breast cancers are HER2-negative. HER2-positive alterations occur in 20% of breast cancers [97]. The LANDSCAPE study showed an intracranial response rate of 66% with lapatinib and capecitabine in newly diagnosed radiation-naïve patients [98] with a median duration of response of 5.5 months in HER2+ metastatic breast cancer patients, PATRICIA trial demonstrated an intracranial response rate of 11% with a greater dose of trastuzumab (6 mg/kg) and pertuzumab in patients who did not respond positively to prior trastuzumab as well as radiotherapy. Fifty-one percent of patients achieved clinical benefit at 6 months. Trastuzumab emtansine (T-DM1) has shown efficacy in intracranial disease control. The KAMILLA study of T-DM1 demonstrated the overall response rate as 21% in advanced or metastatic HER2+ breast cancer patients who had received prior HER2-based management along with chemotherapy with no positive effect [99]. The median duration of exposure was 9.5 months. PERMEATE was an investigator-initiated, multi-centric study of pyrotinib plus capecitabine in HER2-positive breast cancer brain metastasis patients of 78 patients, cohort A included 59 patients that were radiation naïve and cohort B included 19 patients that had progressed on radiation. The combination resulted in an IRR rate of 74·6% in cohort A and 42·1% in cohort B [100]. Neratinib in combination with capecitabine has shown intracranial response rates of 33–49% in patients with progressive disease post-radiation [101]. The HER2CLIMB study randomized patients who earlier received trastuzumab, pertuzumab, and T-DM1 to the regimen of tucatinib, capecitabine, and trastuzumab versus capecitabine and trastuzumab alone, recognized a complete intracranial response rate of 47% with tucatinib in brain metastases patients with a median duration of response of 6.8 months; respective guesses in patients just receiving trastuzumab and capecitabine were 3 months and 20%, respectively [102]. The DESTINY-Breast 01 trial demonstrated an overall response rate of 58% and a CNS response rate of 41% in 24 brain metastases patients who were heavily pretreated (median = 6 prior regimens). Additionally, the median duration of response was 18.1 months [103]. Another trial that is ongoing (DESTINY breast-12) will enroll up to 250 patients with stable or progressive HER2+ breast cancer brain metastases to further define the intracranial activity of trastuzumab deruxtecan (T-DXd). Systemic therapy options are limited for patients with triple-negative breast cancer (TNBC) and brain metastases. The ASCENT trial evaluates sacituzumab govitecan in TNBC patients with brain metastases. It is an antibody–drug conjugate comprising of an anti-Trop-2 antibody attached to an active metabolite called irinotecan, SN-38. The clinical benefit rate and intracranial response rate were 9% and 3%, respectively [104]. A current SWOG trial is assessing the CNS activity of sacituzumab govitecan, especially in active brain metastases patients (NCT04647916). Alpelisib, a PI3K inhibitor, may have intracranial efficacy for HER2-negative breast cancer patients, after its use led to improved outcomes in a case series of 4 patients [105] and, Abemaciclib showed a response rate of 5% for HER2 negative and 0% for HER2 positive patients [106]. Efficacy of PARP inhibitors such as olaparib is limited in BRCA mutated breast cancers although there is limited data in brain metastases [107]. Bevacizumab in combination with carboplatin in breast cancer brain metastases patients has shown clinical efficacy, and in a phase II trial, their combination demonstrated a CNS ORR of 63% (95%CI, 46–78), a median PFS of 5.62 months, and an OS of 14.10 months [108]. Eribulin was utilized in the EBRAIM prospective observational trial among patients with HER2-negative breast cancer brain metastases, lead to 14 patients with disease control and a prolonged PFS of 10 months (vs 4 months) [109]. Capecitabine has also shown activity in HER2-negative breast cancer brain metastases, as it demonstrated median OS of 13 months and a PFS of 8 months, among a cohort of 7 patients after capecitabine initiation [110].

Iniparib, a PARP inhibitor which also acts by changing reactive oxygen species metabolism in tumor cells, has been evaluated in combination with irinotecan in patients with TNBC brain metastases, and an intracranial response rate of 12% was reported among 34 evaluable patients [111]. The IMpassion 130 reported the efficacy of atezolizumab with nab-paclitaxel or placebo, for treatment of metastatic TNBC and reported a median OS of 14.3 months in patients with concomitant brain metastases [112]. A phase I study of capecitabine in combination with temozolomide for the management of TNBC brain metastases reported significant antitumor activity, with 1 complete and 3 partial responses leading to an ORR of 18% in the brain [113]. Temozolomide was also assessed in combination with cisplatin, in a phase II study, and lead to six patients with breast cancer brain metastases achieving stable disease [114].

There are several ongoing studies in triple-negative breast cancer brain metastases including atezolizumab in combination with SRS is currently being evaluated in a phase II trial, to evaluate its efficacy in this patient population (NCT03483012). Another phase II trial currently underway is assessing the efficacy of cisplatin in combination with veliparib, another PARP inhibitor; to treat recurrent triple-negative breast cancer–associated brain metastases (NCT02595905). Finally, the CONTESSA TRIO trial which utilized tesetaxel in combination with various PD-L1 inhibitors, among patients with triple-negative metastatic breast cancer, recently concluded, and the intracranial efficacy results are awaited (NCT03952325).

#### Melanoma

The advancement in systemic therapy is beneficial to target actionable mutations (especially BRAF, NRAS) in melanoma patients with brain metastases. The COMBI-MB study studied the effect of dabrafenib and trametinib in patients with the BRAFv600E mutation. The intracranial disease control rate was 75–88% and the median progression-free survival was 4.2–7.2 months as compared to 11.1 months for patients without brain metastasis [115, 116]. Other agents which include BRAF/MEK regimens such as encorafenib and binimetinib or vemurafenib and cobimetinib have shown intracranial activity [117, 118]. More phase I studies are being conducted to evaluate the efficacy of BRAF and MEK inhibitors (NCT04543188, NCT03332589, NCT04190628).

Immunotherapeutic agents such as check-point blockade have shown responses in melanoma brain metastases. Trials with pembrolizumab or nivolumab, yielded only about 20% intracranial response rates as compared to a 35–40% extracranial response rate [119, 120]. Two trials supported dual-agent therapy include Checkmate 204 and the ABC study. CheckMate 204 is a single-arm phase II trial of a combination of ipilimumab and nivolumab in patients with melanoma and active/unirradiated brain metastases. The benefit was primarily seen in asymptomatic melanoma patients with brain metastasis with a clinical benefit rate (CBR) of 58.4%. The symptomatic patients had intracranial CBR of 22.2% and median intracranial progression-free survival of 1.2 months and overall survival of 8.7 months [120–122]. The ABC study is a randomized trial of patients with asymptomatic or unirradiated brain metastases secondary to melanoma assessing the combination of ipilimumab and nivolumab versus nivolumab, along with a single-arm cohort of patients with advanced disease after local therapy, leptomeningeal disease, or neurologic symptoms accomplished with nivolumab monotherapy [8, 120–122]. The trial confirmed superior results with the combination of nivolumab and ipilimumab as compared to nivolumab monotherapy. The PFS at 6 months was 50% for nivolumab plus ipilimumab versus 29% for just nivolumab. The OS at 6 months was 76% for nivolumab plus ipilimumab as compared to 59% for nivolumab alone. Interestingly, patients who had progressed on prior BRAF inhibitor therapy did not have a meaningful result on the combination therapy [120]. This has led to incorporation into the ASCO-SNO-ASTRO guidelines for immunotherapy in NSCLC patients with asymptomatic brain metastases [38]. The use of multi-modality therapy has been shown to be useful in retrospective series compared to either drug alone of radiosurgery alone [123]. Surgery can remove tumor mass decreasing the need for steroid use [100]. Studies focusing on BRAF-targeted and immunotherapeutic approaches are going on (NCT04511013).

## Summary

Significant challenges such as diverse patient populations, selection bias, the efficacy of past treatment, patient dropout, and ambiguity in finalizing primary endpoints in addition to FDA approval for novel systemic therapies exist even though progress has been observed in novel agents in brain metastasis. There has been greater awareness to include brain metastases patients in clinical trials and also novel drugs are being developed with the intent to have intracranial efficacy which has led to substantial progress in management in the last decade. Current endeavors such as reducing patient heterogeneity by utilizing molecular profiling to understand the genetic makeup of the patient’s tumor, broadening eligibility criteria to increase diverse demographic enrollments, particularly of ethnic minorities to understand tumor’s biology, and advancements in the conduct of clinical trials have the potential to improve outcomes for this increasingly important cohort of patients. Additional trials with different drug combinations or with radiation are needed to further improve outcomes.

## Supplementary Information

Below is the link to the electronic supplementary material.

Supplementary file1 (DOCX 35 kb)

Supplementary file2 (DOCX 41 kb)

Supplementary file3 (DOCX 36 kb)

Supplementary file4 (DOCX 43 kb)

Supplementary file5 (DOCX 31 kb)

Supplementary file6 (DOCX 37 kb)

## Abbreviations

N: Number
CR: Complete response
PR: Partial response
SD: Stable disease
PD: Progressive disease
ORR: Objective response rate
OS: Overall survival
PFS: Progression-free survival
